# Transforming poultry production with smart circular sustainability: Bridging digital innovation, circular economy, and risk management for long-term resilience

**DOI:** 10.1016/j.psj.2026.107106

**Published:** 2026-05-09

**Authors:** Farid S. Nassar

**Affiliations:** Department of Animal and Fish Production, College of Agricultural and Food Sciences, King Faisal University, Al-Ahsa, Saudi Arabia

**Keywords:** Circular economy, Digital technology, Poultry production, Risk management, Smart circular sustainability

## Abstract

The global poultry industry is facing increasing challenges and risks, including growing demand, climate change, resource depletion, and stringent regulations, alongside epidemic diseases, market volatility, and supply chain disruptions. These pressures necessitate a fundamental transformation toward more efficient, resilient, and sustainable production systems, highlighting the need for an integrated model that combines Circular Economy (CE), digital technologies, and risk management. Within this context, Smart Circular Sustainability (SCS) emerges as a comprehensive framework that integrates CE strategies, modern digital technology tools, and risk management, aiming to optimize resource use, reduce waste, and enhance productivity while ensuring environmental and economic resilience. This concept does not merely focus on incremental improvements; rather, it seeks to fundamentally restructure poultry production systems into intelligent, adaptive, and predictive systems capable of anticipating disruptions and effectively responding to complex uncertainties, while supporting animal welfare and ensuring long-term food security. This study aims to establish the concept of SCS in the poultry sector and to identify its practical implementation strategies. Methodologically, the study adopts a qualitative descriptive design supported by a systematic literature review conducted in accordance with PRISMA guidelines, integrating peer-reviewed studies, international reports, and global case studies to ensure scientific rigor and analytical comprehensiveness. The findings highlight the need for a unified framework that integrates resource management efficiency, waste reduction, and risk management within a single holistic model to improve economic, environmental, and social dimensions. The study also presents structured strategic pathways for transitioning from traditional production systems to smart, circular, technology-driven systems, and risk management. The current study recommends adopting SCS as a strategic framework to guide production policies in the poultry industry, with an emphasis on low-cost, locally adaptable digital technologies, capacity-building programs, and enhanced multidisciplinary collaboration among academia, industry, and policymakers. Ultimately, this concept represents a transformative pathway toward a balanced poultry production system that integrates economic performance, environmental conservation, animal welfare, and sustainable resource governance for the benefit of future generations.

## Introduction

Global poultry meat production is expected to continue its steady growth, reaching 173 million tons of ready-to-cook poultry products by 2034, representing 62 % of the total growth in global meat consumption (OECD/FAO, 2025), compared to 105.8 million tons in 2025 (USDA, 2025). Moreover, the share of poultry in total animal protein consumption has expanded significantly in recent decades and is projected to supply around 45 % of all meat-derived protein by 2034. This sustained growth is driven by poultry’s lower cost, favorable protein-to-fat ratio, superior nutritional profile, and lower environmental footprint compared to red meat. Accordingly, poultry remains the preferred choice for consumers concerned with both cost and sustainability (OECD/FAO, 2025).

In response to the growing global demand for poultry meat and eggs, production systems should prioritize improving efficiency and optimizing resource use to address climate change while ensuring sustainable and equitable food systems aligned with the SDGs. This requires the integration of modern technologies, where Big Data enables advanced decision-making, Artificial intelligence (AI) and deep learning support accurate predictive modeling, and sensing technologies allow precise real-time monitoring of production environments. Collectively, these innovations enhance resource efficiency, reduce losses, improve animal and worker welfare, and lower environmental and social impacts, thereby establishing a smart, resilient, and sustainable poultry production system capable of effectively addressing future challenges (Franzo et al., 2023).

Moreover, the Earth is experiencing an escalating environmental crisis driven by human activities, leading to severe climate change, biodiversity loss, and ecosystem degradation (Haines and Patz, 2004). By 2050, agricultural production is expected to decline significantly, accompanied by a sharp increase in global food prices (Hristov et al., 2020). This situation is primarily driven by the prevailing linear economic model of “take–make–dispose,” which accelerates resource depletion and exacerbates environmental degradation (Payne and Kwofie, 2024).

In response to these unsustainable patterns, the Circular Economy (CE) has emerged as an alternative to the linear model by emphasizing reuse, recycling, and repurposing of products at the end of their life cycle (Ellen MacArthur Foundation, 2019). This approach is particularly important in the agricultural sector, as it offers significant opportunities for recovering underutilized resources (Awasthi et al., 2019). Unlike natural ecosystems, which operate in closed loops without generating waste, human activities produce large volumes of waste, including harmful materials such as poultry feathers, which can be repurposed into biodegradable, value-added agricultural materials (Kacorzyk et al., 2025).

In contrast, the concept of sustainability has undergone significant conceptual drift, being widely used without clear definition or measurable criteria. Although the widely cited UN definition emphasizes meeting present needs without compromising future generations, it remains broad and non-operational. This ambiguity has weakened the ability to ensure effective commitment toward sustainability goals related to resource conservation and intergenerational equity (Ben-Eli, 2018).

Moreover, climate change, as one of the risks facing the poultry industry, has a significant impact on this sector, which is highly sensitive to environmental variations, as rising temperatures reduce productivity, increase mortality rates, and lower efficiency, resulting in substantial economic losses in broiler production estimated at approximately USD 2.36 billion annually in the United States alone due to heat stress (Abbas et al., 2025). Thus, the ongoing global climate change necessitates the improvement of poultry management systems to maintain optimal environmental conditions that enhance productivity. Achieving food security based on fair, equitable, and sustainable food production systems that utilize modern automation technologies is essential for all countries in order to achieve the United Nations Sustainable Development Goals (SDGs), which include poverty eradication, zero hunger, good health and well-being, industry innovation, and infrastructure (Chigwada et al., 2022).

This study aims to introduce and establish the concept of Smart Circular Sustainability (SCS) in the poultry industry as an innovative framework that integrates CE principles, modern digital technologies, and risk management, with the objective of enhancing production efficiency and achieving sustainable, resilient, and long-term production systems, in addition to identifying the strategies required for implementing this concept within the poultry sector. In this context, The study aims to address four key aspects: first, clarifying the concept of CE and the potential for integrating its principles into the poultry industry to reduce waste and maximize the utilization of by-products; second, highlighting the role of smart technologies in developing a more efficient and sustainable poultry industry; third, defining SCS within the context of poultry science and distinguishing it from both the smart circular economy concept and conventional sustainability; and fourth, presenting the strategies for implementing SCS in the poultry industry, along with the main challenges that may hinder its implementation.

In light of the rapidly escalating environmental and economic challenges facing the global poultry sector, there is an urgent need for an integrated framework that combines technological intelligence, operational efficiency, and long-term sustainability. Despite the growing interest in sustainable livestock production systems, a clear research gap persists in the lack of integration of digital technologies, CE principles, and risk management within a unified SCS framework in the poultry sector. Previous studies have addressed these dimensions separately, leading to fragmented approaches that were unable to capitalize their synergies, despite evidence that their integration can enhance efficiency, strengthen predictive capabilities, reduce emissions, and improve system resilience, particularly in developing contexts characterized by weak digital infrastructure and limited data readiness. Accordingly, this study aims to bridge this gap by developing an integrated conceptual framework for SCS in the poultry sector, combining digital technologies, CE principles, and risk management. This framework enables the development of intelligent production systems capable of prediction, adaptation, and proactive decision-making in response to environmental and economic changes, while also proposing an implementation model that enhances transparency, data-driven governance, and the long-term sustainability of food security systems.

## Material and methods

**Fig. 1:**
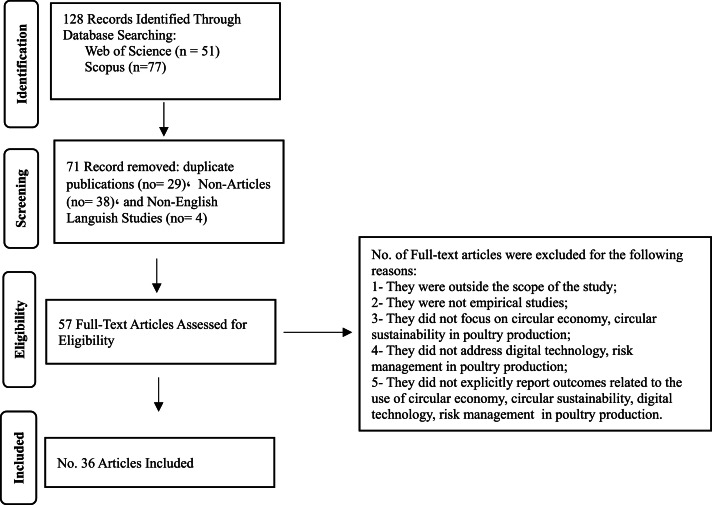
PRISMA flowchart of study selection and inclusion process.

This study employs a qualitative descriptive research methodology to introduce and establish the concept of Smart Circular Sustainability (SCS) in the poultry industry, as an innovative framework that integrates the principles of the Circular Economy (CE), modern digital technologies, and risk management in order to enhance production efficiency and achieve resilient, long-term sustainable production systems. The study followed the PRISMA guidelines (Liberati et al., 2009; Moher et al., 2015) due to their capacity to enhance transparency, minimize bias, and ensure a systematic and traceable approach to conducting systematic reviews (Afrifa et al., 2022), with the methodological steps clearly illustrated in Fig. 1. To enhance transparency and methodological rigor, the manuscript has been revised to provide a comprehensive PRISMA flow description. A total of 128 records were initially identified through database searching, including Web of Science (*n* = 51) and Scopus (*n* = 77). Following the initial screening, 71 records were excluded, comprising duplicate publications (*n* = 29), non-article documents (*n* = 38), and non-English language studies (*n* = 4). Additional exclusion criteria included studies that were outside the scope of the research, non-empirical in nature, not focused on CE or circular sustainability in poultry production, and those that did not address digital technologies or risk management within poultry production. Furthermore, studies that did not explicitly report outcomes related to the application of CE, circular sustainability, digital technologies, or risk management in poultry production were also excluded. Subsequently, 57 full-text articles were assessed for eligibility, of which 36 articles met the predefined inclusion criteria and were ultimately included in the review, as clearly presented in Fig. 1.

This study adopted a structured four-phase research methodology designed to enhance methodological rigor and ensure analytical integration and precision. Phase one, involved conducting systematic and comprehensive search across major academic databases, including Scopus and Web of Science, using a set of precise keywords such as "Poultry Production", "Poultry Industry", “Circular Sustainability”, “Circular Economy”, “Digital Technology”, “Digital Innovation", and "Risk Management". The search focused on peer-reviewed articles, institutional reports, and empirical studies published in English for the years 2015 and 2025, supplemented with foundational literature to ensure both the breadth and depth of the theoretical framework. phase two: established clear inclusion and exclusion criteria to ensure analytical validity and reliability. Studies were included if they addressed the concepts of sustainability, CE, digital technologies, risk management and their impact on poultry production. Studies lacking empirical evidence, methodological rigor, or direct relevance to poultry production settings were excluded to maintain analytical clarity and consistency.

Moreover, Phase three: focused on qualitative data extraction and analysis, examining each study’s objectives, key findings, and implications of applying sustainability principles, CE models, digital innovations, risk management within the poultry sector. This phase moved beyond descriptive review toward thematic synthesis, integrating findings across multiple sources to identify common patterns, differences, sectoral vulnerabilities, and critical intervention points, thereby adding analytical depth and intellectual originality to the study’s outcomes. In phase four, involved an in-depth synthesis and interpretation of the collected findings to explore how the poultry industry can be restructured through an integrative framework that merges traditional sustainability, CE, digital technologies, and risk management under the unified concept of SCS. This phase expanded the analysis from description to strategic application, proposing practical strategies for implementing SCS within the poultry sector to enhance production efficiency and develop resilient, long-term sustainable production systems through continuous collaboration among key stakeholders, including poultry science programs in higher education institutions, government agencies, and commercial poultry producers.

By integrating descriptive, interpretive, and strategic dimensions within this four-phase methodological framework, the study provides a rigorous and comprehensive scientific evaluation of how sustainability, CE, digital technologies, and risk management are being integrated into the development of the poultry industry. This framework addresses previous research gaps and offers evidence-based strategies to promote the transition toward SCS, achieving greater production efficiency, stronger food security, and more resilient and sustainable production systems, while maintaining the credibility, scientific value, and practical relevance of the research in supporting the future sustainability of the poultry industry

## Results and discussion

### Circular economy concept and its integration into the poultry industry to minimize waste and maximize by-product utilization

#### Concept of the circular economy

Ensuring the production of sufficient, safe, and nutritious food for future generations without exhausting resources or damaging ecosystems remains a critical global challenge (Rockström et al., 2017). Modern agriculture increasingly depends on external inputs, including synthetic fertilizers, irrigation, imported feed, agrochemicals, and infrastructure. While these inputs can boost productivity, they also risk generating waste and contributing to environmental degradation. CE based business models offer strategies to mitigate these pressures (Purnell et al., 2019). Although no single universally accepted definition of CE exists, its core principles focus on eliminating waste and pollution, maintaining products and materials in continuous use, and regenerating natural systems (Ellen MacArthur Foundation, 2019).

The principle of eliminating waste and pollution emphasizes the adoption of renewable and sustainable inputs, including substituting primary resources with products derived from waste and prioritizing locally sourced inputs whenever feasible (Murray et al., 2017). The principle of maintaining use focuses on extending the lifespan of products and materials to reduce the demand for new resources. Related concepts include product longevity (Figge et al., 2018), responsible product stewardship, and business models that facilitate resource redistribution (Rizos et al., 2016). Additionally, Murray et al. (2017) highlights the importance of designing and managing systems that support both ecosystem functions and human well-being.

Moreover, CE is built upon principles aimed at minimizing waste and maximizing resource efficiency, emerging from the imperative to safeguard the planet’s finite natural resources. Traditionally, societies extracted and utilized materials with little regard for the environmental consequences of their disposal. CE addresses these impacts by implementing strategies to mitigate potential risks, emphasizing the sustainable integration of economic activities with environmental well-being. Its core focus lies in redesigning material cycles and processing systems, enhancing ecosystem services, and promoting human well-being (Valavanidis, 2018). By conserving resources, CE fosters economic growth (Stahel, 2016) and tackles challenges related to resource scarcity and waste management by recovering economic value from waste streams (Homrich et al., 2017). Recognizing these sustainability benefits, governments worldwide actively promote, and in some cases mandate, the adoption of CE principles and practices (UNIDO, 2017).

The concept of “closing the loops” in arid regions is grounded in economic models that emphasize recycling and reuse (Voulvoulis, 2018). Additionally, CE initiatives, therefore, promote environmentally sustainable practices such as employing recyclable packaging and prioritizing eco-friendly products. These initiatives contribute to reducing energy consumption, minimizing waste generation, and mitigating harmful environmental impacts while eliminating unnecessary materials (Ghisellini et al., 2016). By adhering to the principle of “closing the loops,” CE offers a pathway to move away from the linear production model, which produces significant waste and carbon emissions, toward a low-waste, carbon-neutral economy (Scarpellini et al., 2017).

The most formal, though still debated, definition of CE was presented by Kirchherr et al. (2017): “CE describes an economic system based on business models that replace the concept of ‘end-of-life’ with reducing, reusing, recycling, and recovering materials in production/distribution and consumption processes, thus operating at the micro (products, companies, consumers), meso (eco-industrial parks), and macro (city, region, nation, and beyond) levels, with the aim of achieving sustainable development, which entails creating environmental quality, economic prosperity, and social equity to the benefit of current and future generations.”

A necessary and sufficient definition of CE should encompass closed loops, optimal resource use within the system, and multi-level resource cycles, while acknowledging the imperfection of recycling processes due to the laws of thermodynamics. This led Figge et al. (2023) to propose an alternative definition stating: “CE is a multi-level resource-use system that stipulates the full closure of all resource loops. Recycling processes and other means that improve the scope and direction of resource flows contribute to supporting CE as enabling practices and activities. In its ideal conceptual form, all resource loops are completely closed. In its imperfect real-world form, however, the use of some virgin resources remains unavoidable.”

In addition to, CE has emerged as a leading global paradigm, prioritizing reuse, recycling, and waste reduction over the traditional linear model of extraction, consumption, and disposal. The integration of information and communication technologies, including Big Data, Cloud Computing, and IoT, is driving a digital transformation that reshapes ecosystems, business models, and technological infrastructures in support of CE. The expanding capabilities of IoT, combined with CE principles, have fostered new technological frameworks and circular design strategies. Key research areas focus on IoT-enabled systems for reuse, remanufacturing, and recycling, as well as the challenges involved in their implementation (Voulgaridis et al., 2022).

In recent years, CE has gained significant attention among policymakers, businesses, and academic institutions. Governments are advancing the CE agenda through both regional and national initiatives, particularly within the European Union and China. Notably, the European Green Deal, which seeks to achieve climate neutrality, includes policies aimed at shifting the economy from a linear to a circular model to reduce waste generation, including in the food sector, and mitigate environmental degradation (Almulhim, 2024).

Within agricultural and food systems, CE is more accurately described as the circular bioeconomy, distinguishing it from circular models in manufacturing sectors that depend largely on non-renewable, extracted materials. Additionally, the notion of CE in agriculture is frequently conflated with “farm-to-fork” frameworks and supply chain accountability, which emphasize life-cycle management and product traceability extending both upstream and downstream of the farm gate. Such frameworks are, however, predominantly shaped by the context of high-income countries (Duncan et al., 2023).

Conversely, CE is increasingly recognized as a strategic framework for advancing SDGs. Its relevance to the agricultural and Agri-food sectors is particularly notable, given that these systems are inherently based on biological cycles and generate significant amounts of waste across the entire supply chain. Moreover, the sector presents distinctive opportunities to foster circularity, especially through its broad capacity to transform biological waste into valuable inputs for new production processes (Rukundo et al., 2021).

From the foregoing, it is evident that CE in the agricultural and food sector represents an advanced strategic framework for redefining the relationship between production, consumption, and natural resource management, by converting waste into productive inputs, extending the lifespan of resources and products, and maximizing their efficiency through closed loops and multiple levels. The implementation of this concept goes beyond merely reducing waste and mitigating environmental impact; it also enhances ecosystem functions, supports human well-being, and achieves comprehensive economic and social sustainability, particularly through the adoption of technological innovations such as IoT, Big Data, and Cloud Computing, which enable the design of intelligent systems for resource management, tracking, and improved reuse and recycling. Furthermore, CE strengthens resilience to environmental and economic challenges such as resource scarcity, carbon emissions, and soil degradation, while creating new economic opportunities by utilizing organic waste in new production processes, making it an effective tool for achieving SDGs. In the agricultural context, especially in the production of both animal and plant-based food, CE provides a practical model for reducing reliance on non-renewable inputs, recycling biological waste, and achieving sustainable integration between productivity and environmental efficiency, underscoring that the transition from the traditional linear system to CE is not merely an option but a strategic necessity to ensure food security and preserve ecosystems for current and future generations.

#### Integration circular economy principles into the poultry industry to minimize waste and maximize by-product utilization

In poultry industry, as one of the largest sectors in global food production, faces increasing pressures related to resource scarcity, environmental degradation, and waste management. Traditional production models generate substantial amounts of by-products, such as feathers, litter, and processing residues, which are often discarded or underutilized. Integrating CE principles into poultry production provides a strategic approach to transform these by-products into valuable resources, extending the lifecycle of materials, and enhance overall system efficiency. By adopting practices such as recycling, reuse, and waste valorization, alongside leveraging technological innovations, the poultry sector can reduce environmental impacts, improve economic returns, and contribute to sustainable food production for current and future generations.

Campos et al. (2020) demonstrated the potential utilization of poultry industry by-products, such as poultry fat, poultry by-product meal, and hydrolyzed feather meal, as alternative ingredients to conventional fish meal and fish oil in aquaculture feeds. This approach contributes to waste reduction and enhances overall sustainability. Moreover, Grassi et al. (2023) reported that poultry slaughterhouse sludge can be effectively converted into activated carbon capable of removing pharmaceutical contaminants from water. The results showed exceptionally high efficiency compared to conventional carbon, with strong pollutant removal capacity and the ability to be reused multiple times while maintaining performance, making it a sustainable solution for poultry waste management and environmental protection.

Building on this concept of waste valorization, Kacorzyk et al. (2025) found that the use of poultry feather waste for producing biodegradable geotextile materials resulted in more than 91 % mass loss within 24 weeks, a 266 % increase in seedling emergence, and a 90 % improvement in plant growth. It also enhanced soil moisture and microbial activity, confirming its effectiveness as a sustainable solution for soil health improvement and agricultural support.

Similarly, Li et al. (2025a) reported that deep eutectic solvent processing of poultry feather waste achieved a dissolution rate of 79.97 % and an extraction yield of 50.26 % within two hours at 92°C. The process produced highly digestible peptides and released amino acids at a rate of 241.08 µg/mL, representing a 63.9 % increase compared to fish meal, highlighting its efficiency in converting feather waste into high-value feed protein within a CE framework. In the same direction of maximizing resource recovery, Stiborova et al. (2020) noted that poultry meat production and consumption have doubled over the past 17 years, and since only 70 %–80 % of the carcass is utilized, substantial waste is generated, particularly from mechanical separation processes. Advanced methods have been developed to convert these wastes into bio-based products used as functional feed ingredients, thereby reinforcing sustainability principles and CE applications.

Moving from material recovery to energy generation, Cé et al. (2025) confirmed that turkey litter can serve as a promising feedstock for biogas production, generating approximately 93.4 ± 4.6 standard liters of methane per kilogram over 50 days, with an estimated annual energy yield of 2.43 × 10⁴ MWh and a reduction of approximately 34,000 tons of CO₂ equivalent, thereby supporting renewable energy production and reducing environmental impact. Likewise, a study conducted in Malaysia showed that poultry waste utilization for biogas and solar energy systems could generate 27,452.04 kWh per biogas cycle with an excess of 15,522.04 kWh, in addition to 12,000 kWh/month from a 100-kW solar system covering 25 % of broiler houses rooftops. This reduces electricity consumption by 5 % and supports the transition toward a low-carbon CE within poultry industry (Manogaran et al., 2025).

In this context, integrating poultry waste management within CE framework represents an effective pathway toward sustainability, enabling the conversion of waste into bioenergy and high-value organic fertilizers that enhance soil fertility and reduce reliance on chemical inputs. It ensures optimal resource utilization, minimizes waste generation, and contributes to ecological balance. Economic assessments further confirm that these technologies are financially viable for medium- and large-scale farms, offering significant economic returns (Kocetkovs and Zvirbule, 2025). In countries with high poultry production, solid organic waste represents a major environmental and economic challenge. The production chain generates large quantities of litter, feed residues, carcasses, and sludge, with limited recovery of nutrients and energy. Composting is an effective solution, optimizing the carbon-to-nitrogen ratio using plant-based materials such as tree pruning residues, cotton waste, crude glycerin, and Napier grass, resulting in organic fertilizer that supports circular production systems (Chiarelotto et al., 2021).

In parallel, the expansion of the poultry industry has also increased feather waste, which remains underutilized due to limited industrial applications. Producing biodegradable nonwoven fabrics from feathers offers a sustainable solution that improves soil properties, enhances plant growth and microbial activity, and serves as an eco-friendly alternative to conventional synthetic materials while supporting nutrient recycling (Kacorzyk et al., 2025). Additionally, eggs are among the most widely consumed animal products globally; however, the disposal of spent laying hens after their productive cycle poses environmental challenges, as they are often landfilled or incinerated, leading to greenhouse gas emissions and resource loss. Studies indicate that more than 13,948 tons of protein can be recovered from viscera, feathers, and blood, and that spent hens can be reused for human consumption or converted into energy through anaerobic digestion or microbial fermentation (Amicarelli et al., 2023).

Ultimately, effective waste management in the poultry industry is essential for achieving environmental and economic sustainability. Additionally, CE enables the transformation of waste into valuable resources, such as bioenergy, organic fertilizers, and biodegradable feather-based fabrics, enhancing soil fertility and reducing reliance on chemical inputs. Composting and reusing residues, including litter, feed remains, and carcasses, support closed-loop strategies that increase resource efficiency and minimize environmental impact. Managing spent laying hens through protein recovery or anaerobic digestion further optimizes resource use. Overall, integrating technological innovations, sustainable farming practices, and supportive policies is crucial to transition from linear models to CE systems, ensuring sustainable production, environmental protection, and long-term socio-economic benefits.

### The role of smart technologies in enhancing production efficiency and their relationship to achieving sustainability in the poultry industry

#### Smart technologies and enhancing production efficiency in the poultry industry

The generation of Big Data has become a fundamental feature of modern agriculture, offering significant opportunities to improve farm profitability, reduce environmental and social impacts, and enhance human and animal health and welfare through the integration of data collection technologies, computational capabilities, and advanced analytical tools. In poultry farming, various sensing technologies such as optical, acoustic, and wearable sensors, as well as thermal infrared imaging and optical flow analysis, contribute to more precise and efficient farm management. These capabilities are further strengthened by the application of Machine Learning and Deep Learning techniques, which enable the development of highly accurate classification and prediction models, ultimately enhancing the efficiency and effectiveness of agricultural production systems (Franzo et al., 2023).

In poultry farms, data management is a critical factor for increasing productivity and ensuring animal welfare, particularly within the framework of Industry 4.0. Modern systems rely on sensors and real-time analytics to monitor environmental conditions and production indicators such as ammonia and carbon dioxide, with data transmitted to cloud-based platforms to enhance accuracy and support decision-making. This smart approach contributes to sustainability, improves operational efficiency, and reduces environmental impact (Bumanis et al., 2022). Additionally, the integration of AI and robotics in the poultry industry has significantly enhanced flock health and productivity, as machine learning technologies enable early disease detection and limit disease spread. Sensors and cameras embedded in robotic systems provide continuous monitoring of farm environments and improve feeding and egg collection processes while reducing labor costs. This integration of automation and intelligent analytics enhances operational efficiency and achieves higher sustainability and profitability, despite challenges related to cost and implementation, highlighting the importance of continued research and development in this field (Taleb et al., 2025).

Moreover, studies also indicate ongoing efforts to design prototypes for applying digital technologies in small and medium-sized farms. A smart IoT-based system has been developed for small poultry farms to monitor and control environmental conditions such as temperature, humidity, and air quality using sensors and an internet-connected control unit, with real-time alerts and lighting control to optimize rearing conditions, reduce mortality, and improve production efficiency, proving effective in achieving operational objectives (Orakwue et al., 2022). Similarly, a low-cost IoT-based system for remote poultry farm management has been developed to support small and medium-scale farms, especially in developing countries. It enables real-time monitoring and control of environmental factors such as temperature, humidity, water availability, ammonia levels, and lighting, while providing automated lighting schedules and remote control via smart devices. This contributes to reducing costs, improving production efficiency, increasing egg yield, and enhancing energy efficiency (Chigwada et al., 2022).

With the increasing need for using digital technology in large-scale poultry farms, traditional methods of environmental and poultry health monitoring, which often rely on human expertise, face challenges related to low efficiency and poor real-time performance. Li et al. (2025b) stated that the use of automation systems and inspection robots for detecting beak deformities achieved an accuracy of 92.7 %. In addition, Digital Twin (DT) demonstrated high stability in practical applications, enabling effective linkage between physical conditions and virtual environments, thereby providing an innovative solution for digital transformation in poultry farming (Li et al., 2025b).

Additionally, smart poultry systems have proven effective in predicting egg production, where machine learning models outperform traditional approaches when integrating environmental variables such as ammonia, carbon dioxide, and temperature which enhances prediction accuracy, supports proactive farm management, enables early problem detection, reduces losses, and improves production efficiency (Bumanis et al., 2023). Moreover, Ahmed et al. (2021) demonstrated that machine learning models trained on both synthetic and real datasets can accurately distinguish between healthy and diseased birds with an overall accuracy of 97 %, while comparative analysis of different classification models identified the most effective approach, culminating in a real-time IoT-based predictive framework capable of accurately classifying poultry health for industrial applications.

In the context of organic poultry production, which requires farmers to rely exclusively on organic feed and avoid growth promoters and chemical additives despite uncertainties in production outcomes, with the aim of improving performance, maximizing returns, and reducing losses, research has shown that machine learning technologies represent an effective decision-support tool, achieving prediction accuracy of up to 98.96 % in forecasting production performance (Lastanto and Djatna, 2022).

It is evident that the shift toward modern digital technologies in the poultry industry is no longer a simple operational upgrade, but a comprehensive transformation of farm management systems based on data and digital technologies. The integration of Big Data, the Internet of Things (IoT), advanced sensors, machine learning, and DT technologies enables continuous and precise monitoring of the farm’s internal environment, improving control over key factors such as feeding, ventilation, air quality, and production efficiency, thereby enhancing daily operational performance and reducing waste. This transformation also strengthens the integration between operational efficiency and sustainability by optimizing the use of resources such as feed, energy, and water, reducing operational costs, and improving animal welfare and product quality. It further enhances the farm’s ability to manage operations in a more intelligent and responsive manner by converting operational data into immediate, data-driven decisions. Accordingly, these technologies form a foundational pillar for building more efficient, stable, and sustainable poultry production systems with highly precise and flexible resource management at the farm level.

#### Smart technologies and risk management in the poultry industry

Recent studies consistently highlight that risk management has become a cornerstone for the sustainability and competitiveness of the poultry sector. Díaz Mónica et al. (2024) emphasize that cost control, performance measurement, and the adoption of risk management practices are essential tools for enhancing profitability, ensuring sustainability, and strengthening competitiveness. Adeyonu et al. (2021) further stress that risk management policies must be tailored to farmers’ socio-economic characteristics and risk perceptions to ensure sectoral growth and prevent collapse. In the same vein, Moura et al. (2025) underline the importance of increasing farmers’ risk awareness as a key driver for improving production efficiency and sustainability.

At the operational level, Padilla et al. (2021) demonstrate that risk management plays a critical role in reshaping production decisions according to farm size in response to input price volatility, highlighting strategies such as forward contracting and increasing batch sizes to mitigate economic shocks. Meanwhile, Roy et al. (2024) reveal that the COVID-19 pandemic exposed significant vulnerabilities in global supply chains, underscoring that building sustainable and resilient supply systems requires a comprehensive restructuring of existing strategies. Collectively, these findings illustrate that risk management is no longer an auxiliary option but a strategic framework essential for ensuring the stability and long-term sustainability of the poultry sector in the face of escalating uncertainties and crises.

On the other hand, Collineau et al. (2020) demonstrated that the implementation of specific broiler production practices, such as enhanced cleaning and disinfection between production cycles, exclusive use of air-chilling technology, and strict control of storage conditions and retail temperatures, can significantly reduce the risk of *Salmonella Heidelberg* by 26 %, 34 %, and 88 %, respectively, thereby serving as effective risk management tools for safeguarding public health. Similarly, Rombolà et al. (2024) emphasized that effective avian influenza risk management requires the integration of multi-criteria spatial analysis to identify high-risk areas and guide surveillance activities, in conjunction with the rigorous implementation of biosecurity measures in intensive poultry production systems, while highlighting the critical importance of continuous data updating and evidence-based preventive approaches to mitigate outbreaks and protect both poultry flocks and public health. Additionally, Salvador et al. (2020) indicated that effective crisis management of highly pathogenic avian influenza fundamentally depends on risk-based surveillance and spatial modeling at the farm level, enabling the accurate identification of high-risk zones and the timely implementation of targeted preventive and control measures, thereby enhancing outbreak containment and minimizing its impacts on the poultry sector and public health.

In this context, Big Data analytics and AI play a pivotal role in improving climate prediction capabilities by analyzing vast amounts of data and identifying complex patterns with high accuracy, thereby enhancing future scenario forecasting and supporting more effective adaptation and mitigation decisions. Integrating these tools with physical models further increases prediction accuracy and contributes to the development of more sustainable and resilient climate strategies (Ukoba et al., 2025).

With the accelerating intensification of livestock and poultry production, digital management has become an urgent necessity, and the adoption of DT systems is emerging as a promising solution (Jia et al., 2025; Nassar and Abbas, 2025a). Although current DT applications mainly focus on monitoring farm environments and animal behavior, they hold broader potential, including growth prediction, feed consumption estimation, and supply chain optimization. However, their effectiveness still requires further validation in real-world field conditions. Therefore, future expansion of their use in livestock management requires collaborative research to develop advanced machine learning and deep learning models that maximize their capabilities (Hasan et al., 2024).

Ultimately, it can be concluded that the use of digital technologies in poultry production is not limited to real-time operational management such as feed, water, and animal welfare, but extends to predictive capabilities related to climate change, operational disruptions, input price volatility, and supply chain dynamics across the poultry and related food sectors. This integration ultimately enhances the resilience of the entire sector, enabling it to anticipate and respond effectively to future crises while ensuring long-term sustainability, efficiency, and food security.

### The concept of smart circular sustainability in poultry science and its relation to smart circular economy and traditional sustainability concept

#### Concept of sustainability

Since the concept of sustainable development emerged roughly two decades ago, it has undergone significant evolution, with its definitions expanding and interpretations multiplying, resulting in varying meanings depending on the adopting perspective or organization. This diversity has undermined the concept’s credibility, raised questions about its practical applicability, and even cast doubt on the real value of achievements attributed to it, thereby impeding the intended progress in environmental and social domains, which constitute the foundation of sustainable development (Johnston et al., 2007). Moreover, the growing prominence of sustainable development has been driven by the increasing number of related terms and heightened awareness of sustainability’s importance. Various authors and institutions employ multiple definitions for concepts such as green chemistry, cleaner production, and pollution prevention, prompting research into the ambiguity and classification of these terms. The proliferation of information sources has further contributed to the widespread use and multiplication of sustainability-related definitions, leading to the introduction of new terms and the expansion of existing ones, often without sufficient linguistic or conceptual precision. This multiplicity of definitions has caused significant confusion, as many terms are used with vague, overlapping, or only slightly differing meanings (Glavič and Lukman, 2007).

While the significance of “sustainability” is widely recognized, substantial challenges persist in comprehending and applying the concept in practical settings (Morelli, 2011). Consequently, achieving a clear understanding of sustainability is regarded as a major challenge in implementation science, largely due to the absence of consistent definitions in the literature, as most implementation studies fail to provide a clear definition of sustainability, even when it is being assessed (Moore et al., 2017).

According to Brundtland et al. (1987) sustainable development is defined as “development that meets the needs of the present without compromising the ability of future generations to meet their own needs.” This principle underpins the formulation of the seventeen SDGs adopted by the United Nations in 2015, which aim to promote prosperity for all while safeguarding the planet. Research continues to advance methods and metrics for assessing sustainability (Hacking and Guthrie, 2008; Singh et al., 2009). Broadly, sustainability is commonly understood to encompass three primary dimensions: environmental, social, and economic (Svensson and Wagner, 2015; Khan et al., 2016).

The environmental dimension pertains to the life-support systems that are essential for human survival (Goodland, 1995). As outlined by Daly (1990), it is guided by key principles: the consumption of renewable resources should not surpass their regeneration rate, pollution should remain within the environment’s assimilative capacity, and the depletion of nonrenewable resources should not exceed the development rate of renewable substitutes. These principles necessitate the establishment of clear limits on human activities. The planetary boundaries proposed by Rockström et al. (2009) and refined by Steffen et al. (2015) define globally recognized environmental thresholds that should guide human actions. This dimension also includes aspects such as land use, waste management, public health, and energy and water consumption (Delai and Takahashi, 2011; Khan et al., 2016).

The social dimension, which has received comparatively less emphasis, centers on the preservation of social capital, including community values and norms. According to Goodland and Daly (1996), social sustainability can be promoted through active community participation and the strengthening of civil institutions. Sustaining social capital requires investment in education and other essential areas, as neglect can lead to gradual decline (Goodland, 1995). This dimension also underscores the relationships between businesses and stakeholders, as well as aspects of health, safety, and social responsibility (Delai and Takahashi, 2011; Khan et al., 2016). The economic dimension emphasizes the preservation of capital (Goodland, 1995) and pertains to the resource base that supplies both renewable and nonrenewable inputs for production. It includes considerations such as costs, profits, and the creation of new business opportunities (Svensson and Wagner, 2015).

Ultimately, the concept of sustainable development emerged approximately two decades ago and has evolved with the proliferation of definitions and interpretations, leading to confusion in its application and skepticism about its practical value, despite its importance in guiding global sustainability policies such as SDGs. The concept is built on three main dimensions: environmental, which ensures resource conservation and pollution control within planetary boundaries; social, which focuses on social capital, community participation, health, and safety; and economic, which emphasizes the preservation of capital and resources to support productivity and create business opportunities. The primary challenge lies in the ambiguity and multiplicity of definitions, necessitating the unification of concepts and the development of precise measurement tools to ensure the practical and effective implementation of sustainability, thereby securing environmental, social, and economic benefits for both present and future generations.

#### Concept of smart circular sustainability in the context of poultry science and how does it differ from smart circular economy and traditional sustainability concepts

In recent years, the concept of sustainability has increasingly been reduced to a catchphrase, applied across various contexts without a deep understanding of its true meaning, often prioritizing financial performance over environmental and social considerations, and sometimes extending into unrelated domains. While the widely cited definition of sustainable development is theoretically robust, it lacks operational clarity and measurable criteria, and does not involve future generations in defining their own needs, leaving it susceptible to selective interpretation and instrumental use. This shift undermines genuine adherence to sustainability principles as a long-term commitment to protect life, conserve resources, and ensure intergenerational equity (Ben-Eli, 2018).

In contrast, the poultry sector, as a fundamental pillar of food security at both local and global levels, operates within a highly complex environment influenced by disease outbreaks, feeding price volatility, climate change, supply chain disruptions, and shifting consumer demand patterns. These factors directly affect productivity and the stability of food supply systems. The COVID-19 pandemic has revealed the fragility of global supply chains, highlighting the importance of strengthening crisis management, biosecurity measures, and financial risk control. At the same time, climate change imposes additional pressures on production efficiency and product quality, necessitating the restructuring of the sector toward more resilient and sustainable production systems. This transformation relies on smart risk management, advanced supply chain systems, and effective climate adaptation policies, thereby enhancing the ability to anticipate challenges and develop proactive responses that ensure sector sustainability and food security (Nassar and Abbas, 2025b).

Consequently, it has become crucial to reclaim the true essence of sustainability from the multitude of narrow, vague, and non-measurable definitions that are often economically biased and serve merely as rhetorical justifications for limited outcomes that adhere to a “business as usual” approach. Achieving authentic sustainability in the relationship between humanity and the biosphere requires a rigorous, evidence-based interpretation of the concept’s original intent. For this interpretation to be effective, it must inform the development of political instruments, public policies, and institutional decision-making, with a growing emphasis on addressing the root causes of major threats to sustainability rather than merely managing their superficial symptoms (Johnston et al., 2007).

In the context of poultry production, and with the rapid advancement of digital technologies and the increasing reliance on AI in the agricultural sector, it has become essential to reformulate the concept of sustainability in a modern way that integrates technological innovation with CE principles and risk management. This approach aims to enhance resource management efficiency, achieve an effective balance between economic growth and environmental protection, and strengthen social equity, while ensuring the sector’s ability to adapt to future changes and crises. Accordingly, this study proposes the concept of SCS, which integrates traditional sustainability principles, CE, digital technologies, and risk management to achieve operational efficiency and long-term sustainability in the sector. Moreover, SCS can be defined as follows:

“Smart Circular Sustainability is an integrated approach that combines the principles of sustainability, circular economy, digital technologies, and risk management, with the aim of achieving intelligent and efficient resource management, reducing waste, and enhance efficiency and productivity, while ensuring a sustainable balance between economic growth, environmental protection, and social equity. It also strengthens system resilience and the ability to anticipate and effectively respond to risks"

Based on this, the proposed definition presents an advanced conceptual framework that integrates sustainability with CE, digital technologies, and risk management. This framework focuses on the intelligent management of natural and human resources through the use of artificial intelligence, the IoT, DT technology, and Big Data analytics. This integration enhances the ability to effectively manage real-world conditions and achieve maximum possible production efficiency, while also strengthening systems’ capacity to anticipate and respond to risks, thereby improving overall system resilience.

Furthermore, it enhances the poultry sector’s ability to cope with crises such as disease outbreaks, feed price volatility, supply chain disruptions, and climate change by building more proactive and adaptive systems capable of functioning under uncertainty. It also contributes to improving production stability and resource sustainability, increasing operational efficiency, reducing waste, and enhancing production quality, while ensuring inclusiveness and equity in development—particularly by supporting smallholder farmers in developing countries and improving their fair access to technology, resources, and markets. Accordingly, it represents a dynamic model that achieves a balance between economic growth, environmental protection, and social equity within resilient and adaptive production systems.

When compared with the model proposed by Bressanelli et al. (2022), which integrates digital technologies with CE to enhance sustainability and performance, it remains limited in the social dimension and does not explicitly address risk management or system resilience. Similarly, the model proposed by Payne and Kwofie (2024) focuses on improving efficiency and reducing waste without explicitly addressing risks and crises or providing a vision for fundamental system redesign.

In contrast, SCS differs fundamentally, as it offers a more comprehensive framework that integrates sustainability, CE, digital technologies, and risk management into a unified model. It emphasizes proactive risk anticipation, strengthens social equity, supports small-scale producers, and improves animal and worker welfare. This represents a qualitative shift from merely optimizing existing systems to fundamentally redesigning them into more resilient and proactive systems. For example, In Italy, the quantity of fresh meat waste, including poultry meat, across the Agri-food supply chain is estimated to exceed 0.45–0.50 million tons, equivalent to more than 242–268 million euros. In addition, further losses in energy and water are estimated at approximately 435–481 million euros, representing quantities, characteristics, and costs that are not typically captured in conventional financial reporting (Bux and Amicarelli, 2022). Against this backdrop, the application of SCS concept offers a compelling and direct solution. Rather than treating waste merely as an accounting loss, SCS reframes it as a systemic inefficiency that can be identified, managed, and ultimately transformed into value. By integrating digital technologies, SCS enables full visibility of resource and energy flows across the supply chain, uncovering “hidden” costs such as water and energy losses and emissions that remain invisible in traditional reports.

Moreover, embedding risk management within the SCS framework allows for the anticipation of waste hotspots before they occur, such as spoilage during transport or storage, thereby reducing losses proactively rather than reacting to them after the fact. At the same time, circular economy principles within the framework guide the reintegration of these losses, for instance by converting waste into energy or feed, effectively turning economic burdens into valuable resources. Most importantly, SCS aligns economic, environmental, and social dimensions within a single decision-making framework. It ensures that the true cost of resources is neither ignored nor externalized, but fully integrated into strategic choices—ultimately enabling a production system that is more efficient, transparent, and resilient, and far better positioned for long-term sustainability.

In conclusion, SCS represents an advanced model that redefines sustainability in light of continuous global changes by integrating digital technologies, CE principles, and risk management. Its ultimate goal is to build smarter, more resilient, and sustainable production systems capable of operating in complex and volatile environments while ensuring the long-term sustainability of resources.

#### Impact of implementing smart circular sustainability on the poultry industry and dimensions of traditional sustainability

The concept of SCS represents an integrated transformative framework that reconfigures the poultry production system by merging sustainability, CE, digital technologies, and risk management into a single model based on proactivity and resilience, in response to the nature of a sector operating in a highly complex and uncertain environment. In light of the increasing challenges related to food security, climate change, and market volatility, production development is no longer limited to improving operational efficiency; rather, it now requires building intelligent systems capable of anticipating and adapting to risks. In this context, the utilization of artificial intelligence, IoT, DT, and Big Data analytics enables the development of data-driven production systems that combine real-time analysis with predictive intelligence, thereby enhancing the efficiency of natural and human resource management and shifting the sector from traditional operational models to dynamic, intelligent systems oriented toward sustainability and proactive response.

The application of this concept contributes to transforming poultry farms into fully integrated digital systems managed through continuous data streams, where environmental and production variables are monitored and analyzed to predict performance, feed consumption, disease occurrence, and to support immediate evidence-based decision-making. Moreover, the integration of digital technologies with CE practices enhances the re-utilization of waste and its conversion into economically valuable resources, supporting the production of bioenergy, fertilizers, and alternative feeds, and establishing a closed-loop production system that reduces losses and increases efficiency. This transformation is not limited to performance improvement; it also extends to strengthening system resilience and its ability to face crises such as disease outbreaks, supply chain disruptions, and climate variability through predictive models and proactive response strategies.

Economically, this framework provides opportunities to maximize value through improved input efficiency, reduced costs, and enhanced production quality, supported by predictive algorithms and advanced analytics, while simultaneously strengthening smart governance by enabling decision-makers to use digital tools that support both immediate and strategic decisions. It also redefines the role of the farmer from a traditional operator to a data-driven intelligent production system manager capable of dealing with complexity and uncertainty. At the social level, this approach supports principles of equity and inclusiveness by improving smallholders’ access to technology, resources, and markets, enhancing transparency across the value chain, and improving animal welfare as well as workers’ well-being.

More broadly, SCS goes beyond being merely a tool for efficiency improvement or environmental impact reduction to become a development model that re-engineers the relationship between production, environment, and society by integrating digital innovation with circularity and risk management, thereby achieving a sustainable balance between economic growth, environmental protection, and social equity. This system also enables accurate and real-time measurement of environmental, economic, and social performance through digital indicators, supporting evidence-based policymaking and strengthening adaptive management.

Accordingly, the implementation of SCS represents a qualitative shift from improving traditional systems to redesigning them to become more resilient, proactive, and sustainable, capable of operating efficiently in complex and volatile environments, ensuring resource sustainability, enhancing food security, and building a more intelligent and balanced future for the poultry sector.

The implementation of SCS in poultry production marks a strategic turning point in the evolution of modern agricultural systems. By harmonizing science, technology, circularity, and governance, this approach establishes a comprehensive framework capable of balancing economic growth, environmental protection, and social equity. It transforms sustainability from a theoretical construct into a practical, operational strategy that empowers institutions and communities to achieve SDGs efficiently and innovatively. In essence, SCS lays the foundation for a smarter, cleaner, and more fair future for poultry production by linking innovation with responsibility and development with resource protection.

On the other hand, the concept of SCS is a proposed framework in this study that aims to reframe traditional sustainability across its environmental, economic, and social dimensions. It shifts sustainability from a static model based on a relative balance among these dimensions to an integrated dynamic system driven by data, predictive intelligence, and risk management. This framework emerges in response to rapidly evolving changes and conditions of uncertainty, such as feed price volatility, supply chain disruptions, climate change, and disease outbreaks, through the integration of CE principles, digital technologies, and modern governance mechanisms within a single, more flexible and adaptive system. In this context, this framework is expected to bring a fundamental transformation in the nature of traditional sustainability dimensions, and the changes in these dimensions can be outlined as follows:

#### Environmental dimension

The SCS concept is expected to bring a qualitative transformation in environmental resource management by integrating technological efficiency, CE principles, and risk management. Its objective is not limited to reducing emissions or conserving resources; rather, it extends to managing the environment as an interconnected and dynamic system driven by data and predictive analytics, while also accounting for fluctuations in feed prices, supply chain disruptions, and climate change, given their direct influence on production decisions and overall environmental impact. In this context, IoT, and sensor networks enable real-time monitoring of water and energy consumption as well as emissions, while AI applications integrate environmental data with economic, logistical, and climate indicators to translate them into immediate and proactive operational decisions that reduce waste and enhance efficiency. Moreover, SCS contributes to recycling and converting waste into new resources such as bioenergy, fertilizers, and secondary materials, thereby reducing pressure on natural resources and mitigating the effects of market and supply chain volatility, ultimately lowering the overall environmental footprint and improving resource-use efficiency. Accordingly, this concept shifts environmental management from a traditional approach to an interactive and predictive governance system based on digital innovation, comprehensive circularity, and adaptability to uncertainty.

#### Economic dimension

The SCS concept redefines economic growth as a continuous dynamic process of innovation driven by digital intelligence and production circularity, while incorporating a predictive dimension for unstable changes such as feed price volatility, supply chain disruptions, and climate change, as these are direct factors affecting production costs and market stability. Through AI and Big Data analytics, it is possible to enhance value chain efficiency, predict fluctuations in demand and inputs, and reduce waste in essential resources such as feed, water, and energy, thereby strengthening the ability to make proactive economic decisions that reduce risks and increase operational flexibility. At the same time, CE enables the transformation of by-products such as feathers, manure, and processing residues into high-value economic products such as bioenergy, organic fertilizers, and bio-textiles, supporting income diversification and reducing dependence on volatile external inputs. This integration also enhances supply chain resilience and its ability to adapt to sudden crises and disruptions, whether economic, climatic, or logistical. Consequently, this concept evolves into a smart and future-oriented economic model that combines value maximization with waste reduction, transitioning from unstable linear consumption to efficient and predictive resource utilization within a more sustainable and resilient production system.

#### Social dimension

The SCS concept represents a fundamental shift in the notion of equity and well-being from a traditional framework to a model based on digital and cognitive empowerment, where it not only improves quality of life but also enhances individuals’ and communities’ ability to adapt to economic and environmental changes such as feed price volatility, supply chain disruptions, and climate change, through the provision of real-time data and precise performance indicators that support decision-making. Through digital governance tools and intelligent systems, transparency and accountability in resource management are strengthened, while workers, farmers, and local communities—especially in rural areas and developing countries—are empowered to access information and actively participate in planning and decision-making processes, thereby reducing the knowledge and technology gap and supporting the integration of smallholders into modern value chains. Smart technologies also contribute to improving quality of life and enhancing human and animal welfare through safer, more efficient, and proactively responsive production environments, in addition to creating new job opportunities in the green digital economy, which is particularly important in developing countries where agriculture and poultry sectors represent a major source of income and employment. Thus, this concept goes beyond traditional social responsibility to become a flexible participatory knowledge system that enhances human and social capital, fosters innovation, and contributes to building more equitable rural communities capable of resilience and adaptation to uncertainty.

On the other hand, the balance between economic growth, environmental protection, and social equity within SCS framework is achieved through a multi-level governance system based on integration among government, industry, and academic institutions, thereby enhancing the ability to deal with complexity and uncertainty arising from environmental and economic changes such as feed price volatility, supply chain disruptions, and climate change. Governments are responsible for setting policies and regulatory standards and ensuring social equity, in addition to supporting the adoption of digital technologies in production sectors. The industrial sector works on implementing CE principles, employing digital technologies, and managing resources efficiently within more flexible and responsive production systems. Meanwhile, academic institutions contribute by generating knowledge, developing predictive models, analyzing data, and fostering innovation based on AI and advanced technologies. Thus, this balance is not achieved as the responsibility of a single entity, but rather as the outcome of an interactive and dynamic governance system based on data, technology, and innovation, linking policy, practice, and knowledge production, while simultaneously supporting the ability to predict risks and respond to them proactively to ensure the sustainability of production systems.

### Strategies for implementing smart circular sustainability in poultry industry

The implementation of SCS concept in the poultry industry represents a fundamental transformation toward an integrated production system driven by data, prediction, and risk management. It shifts the sector from a traditional reactive model to a smart and proactive framework that combines digital transformation, circular economy principles, and effective governance within a flexible system capable of adapting to uncertainties such as feed price volatility, supply chain disruptions, climate change, and health crises. This framework aims to transform sustainability from a theoretical concept into an operational system based on the integration of government, industry, academia, and civil society, thereby enhancing data-driven decision-making, predictive analytics, and proactive risk management. Ultimately, its objective extends beyond improving operational efficiency to building a resilient and sustainable production system capable of anticipating and managing crises, maximizing resource utilization within SCS framework, and strengthening the strategic role of the poultry sector in global food security. In this context, six key strategies have been identified for implementing this concept in the poultry sector, as follows:

#### Gradual digital transformation for production processes

Digital transformation is considered the cornerstone of implementing SCS, as it enables the shift from traditional systems based on human experience to intelligent data-driven production systems based on predictive analytics and risk management. By integrating IoT, smart sensors, and DT technology, the production environment can be continuously monitored and future performance predicted, including feed consumption, energy efficiency, disease risk, and the impact of price volatility and supply chain disruptions. Additionally, AI technologies integrate operational data with economic and climate data to generate proactive decisions that reduce risks and enhance efficiency. This represents a strategic shift toward smart poultry farms capable of achieving high operational efficiency and adapting to shocks before they occur, while ensuring a balance between environmental and economic sustainability.

Within this framework, stakeholder roles are clearly defined and interconnected. Governments are responsible for providing digital infrastructure and establishing regulatory standards for data exchange and risk management, while poultry science programs in universities and research centers develop analytical and predictive models and smart applications to support data-driven management. The private sector is responsible for implementing these innovations and evaluating their impact on performance and sustainability. This digital transformation leads to reduced waste of energy, water, and feed, increased production efficiency, and strengthened proactive environmental management, forming a strong foundation for developing fully integrated and sustainable digital poultry farms.

#### Adoption of circular economy in resource and waste management

Integrating CE principles into the poultry industry represents a fundamental pillar of SES, aiming to convert waste and by-products into economically valuable resources and energy within closed production loops supported by predictive resource management. Through the establishment of anaerobic digestion units, poultry farms can produce biogas for clean energy generation, while organic waste treatment enables the production of natural fertilizers or raw materials for innovative industries such as bioplastics and alternative feeds. Water and heat recycling also contributes to reducing resource losses and improving overall efficiency.

The application of this circular model leads to reduced carbon emissions and increased resource self-sufficiency within farms, enhancing production resilience and long-term sustainability in the face of challenges such as climate change and resource scarcity. Governments play a key role by providing regulatory frameworks and incentives for farms adopting CE practices, while universities and research centers focus on developing bioconversion and environmental treatment technologies. The private sector, in turn, leads commercial implementation and investment in sustainable value chains. Through this integration, poultry production transitions from a linear system to a circular and synergistic system that improves resource efficiency, reduces emissions, and enhances sector resilience.

#### Building a smart governance and environmental monitoring system

Smart governance represents the regulatory dimension within SCS framework, ensuring the integration of data, sustainability, and risk management into a unified system among all stakeholders (Zhao et al., 2025). This approach focuses on developing unified digital platforms that connect farms, government authorities, and environmental agencies within an integrated data system capable of tracking environmental, economic, and social performance indicators in real time. Smart dashboards play a crucial role in displaying indicators such as supply disruptions, feeding price changes, disease outbreaks, resource consumption, emissions, and animal welfare conditions, enabling decision-makers to take proactive actions based on accurate data.

Smart governance also facilitates the preparation of standardized digital sustainability reports aligned with international standards, enhancing transparency and compliance while supporting risk management at the sector level rather than only at the farm level. This increases consumer and investor trust and ensures compliance with modern environmental and economic regulations. Governments play a supervisory role by setting performance standards, while environmental institutions and universities contribute to designing measurement and analytical systems. Meanwhile, production facilities implement internal smart monitoring systems. This approach strengthens a culture of digital accountability and supports adherence to international environmental standards while enhancing sector sustainability.

#### Capacity building and knowledge empowerment in poultry sector

Human capital development is a critical factor in the success of SCS in the poultry industry, as advanced technologies and intelligent data analysis cannot achieve real impact without a qualified workforce capable of managing complex systems. This approach enhances the role of poultry science programs in universities through specialized curricula integrating smart agriculture, data analytics, CE, and smart farm management, aiming to prepare a new generation of farmers, engineers, and specialists capable of leveraging technology to achieve environmental, economic, and social sustainability. Field training programs and digital learning platforms also contribute to evidence-based decision-making, local innovation, and improved production efficiency.

Universities act as knowledge hubs, governments provide accreditation and policy support, the private sector offers training and employment opportunities, and international organizations facilitate knowledge transfer, particularly in rural communities and developing countries. Thus, human capital becomes a driving force for sustainable transformation in the sector.

#### Green financing and economic incentives

Green financing is a key tool for accelerating the adoption of SCS in the poultry sector by encouraging farmers and investors to invest in environmentally friendly technologies. This includes low-interest loans, tax exemptions, and public-private partnerships to finance digital infrastructure. Governments and financial institutions play a regulatory and facilitative role, while the private sector directs funding toward sustainable production models, and research institutions evaluate environmental and economic impacts. Thus, financing becomes a strategic instrument for environmental and digital transformation.

#### Strengthening research integration and multi-stakeholder collaboration

This strategy focuses on unifying research efforts between universities, industry, and government agencies to accelerate innovation in SES. Through collaboration, DT, and predictive risk management systems can be developed to simulate farm performance and support decision-making. Establishing research networks further enhances knowledge exchange and translates research outcomes into practical applications. Universities provide scientific leadership, governments offer funding and direction, and the private sector implements innovations in practice.

The implementation of SCS strategies represents a radical transformation in the poultry industry from a traditional production model to an integrated intelligent system based on data, prediction, CE, and risk management, thereby reshaping operational mechanisms and decision-making toward greater efficiency and proactivity. This transformation enhances resource efficiency, reduces costs and emissions, and strengthens the ability to adapt to crises and fluctuations in supply chains, input prices, and climate change, supporting long-term food security and sustainability. It also creates new high-value employment opportunities and enhances technical and knowledge-based skills within the sector, establishing a more flexible, intelligent, and predictive production model capable of managing uncertainty and redefining the future of the global poultry industry, making SCS the most effective strategic pathway for its development.

### Key challenges for implementing smart circular sustainability in poultry industry

Recent literature indicates that digital transformation and data management represent the cornerstone for developing the poultry sector, particularly in developing countries that still suffer from clear structural gaps in digital infrastructure and institutional capacities. In this context, Chapot et al. (2024), in the case of Indonesia, highlighted that market and policy volatility, along with weak systems for collecting and managing production and health data, and insufficient coordination among stakeholders, are among the most prominent barriers limiting sector efficiency. The study emphasizes that standardizing data systems, developing data-sharing mechanisms, strengthening training in digital technologies, and supporting research and innovation are essential conditions for improving decision quality within value chains, thereby enhancing productivity and reducing health and economic risks.

Similarly, Sedina et al. (2025) pointed out that accelerating the adoption of digital technologies in the poultry sector in Togo requires integrated policies that go beyond mere technological introduction. Such policies must also include the development of digital infrastructure, human capacity building, and the design of flexible interventions that consider the varying needs of actors across the value chain, from production to marketing. This perspective reflects that digital transformation is not purely a technical process, but rather a systemic one that requires alignment between technology, policy, and socio-economic realities to ensure a meaningful and sustainable impact within the sector.

Furthermore, Olanrewaju et al. (2023), in the case of Nigeria, confirmed that digital technologies hold significant potential for improving the efficiency and productivity of the poultry sector; however, their adoption rates remain limited, particularly due to the dominance of small-scale farms and weak investment capacity. The study indicates that the lack of adequate supportive policies, low technological awareness, and the limited suitability of some digital solutions to the local context constitute major barriers to achieving comprehensive digital transformation. Accordingly, achieving the desired transformation requires redirecting research and development toward more flexible and inclusive digital solutions that consider economic and social dimensions and support the integration of smallholders into the digital ecosystem.

Ultimately, the implementation of SCS framework faces a set of interrelated challenges. Foremost among them is the high cost of digital infrastructure, AI, and DT, which limits the ability of small producers to adopt this transformation and deepens the digital divide within the sector. In addition, poor data quality, fragmentation, and the lack of standardized data collection systems represent a fundamental challenge, as predictive models and risk management systems depend on accurate and integrated data, which are not sufficiently available in many current production systems. Moreover, human and institutional adaptation challenges arise, as this transformation requires rebuilding human capacities and shifting managerial mindsets toward data-driven management, which may face organizational resistance or knowledge gaps, especially in rural contexts. Governance, data security, and privacy challenges also emerge due to the increasing reliance on data exchange among multiple actors, in addition to uncertainty associated with market fluctuations, climate variability, and supply chain disruptions, all of which may reduce the accuracy of predictive models unless they are developed in a flexible and adaptive manner capable of absorbing dynamic shocks.

## Conclusion

Global poultry industry is facing increasing pressures resulting from the growing demand for food, the exacerbation of climate challenges, resource scarcity, and disruptions in supply chains. This makes the continuation of traditional production models insufficient to meet the requirements of food security and sustainability. In this context, this research proposes the concept of SCS as a transformative framework that redefines sustainability within the sector, through the integrated fusion of CE and advanced digital technologies such as artificial intelligence, IoT, DT, and Big Data analytics, in addition to embedding risk management as a core element in the design and operation of production systems. This framework demonstrates that the transition from traditional sustainability to SCS is not limited to improving operational efficiency, but rather represents a comprehensive restructuring of the production system toward a more intelligent, predictive, and adaptive model. The integration of real-time data, predictive models, and CE principles enables increased productivity, reduced waste and emissions, improved resource utilization, and enhanced proactive responses to crises such as feed price volatility, supply chain disruptions, and climate change.

Furthermore, this research confirms that the implementation of SCS strategies not only enhances economic and environmental performance, but also transforms decision-making structures within the sector from reactive management to data-driven smart governance. This supports human and animal welfare, strengthens value chain resilience, and improves food security efficiency at both local and global levels. Ultimately, this work presents a forward-looking vision that establishes a new production paradigm in the poultry industry, based on the integration of technology, circularity, sustainability, and risk management, marking a fundamental shift from traditional production systems to dynamic intelligent systems capable of learning, adapting, and predicting. It also opens broad avenues for future applied research and confirms that adopting this framework represents a strategic step toward achieving SDGs and building a more efficient, resilient, and sustainable poultry sector in a rapidly changing world.

## Funding

This work was supported by the Deanship of Scientific Research, Vice Presidency for Graduate Studies and Scientific Research, King Faisal University, Saudi Arabia (Grant No. KFU253987).

## CRediT authorship contribution statement

**Farid S. Nassar:** Writing – review & editing, Writing – original draft, Visualization, Validation, Supervision, Software, Resources, Project administration, Methodology, Investigation, Funding acquisition, Formal analysis, Data curation, Conceptualization.

## Disclosures

The author declares that there are no conflicts of interest regarding the publication of this paper.
